# Microstructural and Wear Behavior Characterization of Porous Layers Produced by Pulsed Laser Irradiation in Glass-Ceramics Substrates

**DOI:** 10.3390/ma6093963

**Published:** 2013-09-09

**Authors:** Daniel Sola, Ana Conde, Iñaki García, Elena Gracia-Escosa, Juan J. de Damborenea, Jose I. Peña

**Affiliations:** 1 Materials Physics Center, University of Basque Country-CSIC, Pº Manuel Lardizabal 5, San Sebastian 20.018, Spain; 2 Department of Surface Engineering, Corrosion and Durability, National Centre of Metallurgical Research CENIM-CSIC, Av. Gregorio del Amo 8, Madrid 28.040, Spain; E-Mails: a.conde@cenim.csic.es (A.C.); igarcia@cenim.csic.es (I.G.); elenagracia@cenim.csic.es (E.G.-E.); jdambo@cenim.csic.es (J.J.D.); 3 Department of Science and Technology of Materials and Fluids, Material Science Institute of Aragon, University of Zaragoza-CSIC, Maria de Luna 3, Zaragoza 50.018, Spain; E-Mail: jipena@unizar.es

**Keywords:** pulsed laser irradiation, layers, wear resistance, glass-ceramic

## Abstract

In this work, wear behavior and microstructural characterization of porous layers produced in glass-ceramic substrates by pulsed laser irradiation in the nanosecond range are studied under unidirectional sliding conditions against AISI316 and corundum counterbodies. Depending on the optical configuration of the laser beam and on the working parameters, the local temperature and pressure applied over the interaction zone can generate a porous glass-ceramic layer. Material transference from the ball to the porous glass-ceramic layer was observed in the wear tests carried out against the AISI316 ball counterface whereas, in the case of the corundum ball, the wear volume loss was concentrated in the porous layer. Wear rate and friction coefficient presented higher values than expected for dense glass-ceramics.

## 1. Introduction

Laser processing has been incorporated in industry over the last decades being of great interest in the field of optics, electronics, microelectronics, aerospace and medicine. Several methods for processing, such as laser machining, micromachining, marking, drilling and pulsed laser deposition have been developed [1,2]. Laser techniques are cost-effective compared to other traditional surface modification methods and it may be applied to a wide range of substrates, such as metals, ceramics and semiconductors [3]. Reduction in production costs, staff and maintenance savings as well as in tool wearing, make laser processing the most suitable working tool for machining hard and brittle materials.

The appearance of techniques for generating short and ultrashort laser pulses, ranging from tens of nanoseconds to a few femtoseconds, have allowed the availability of more powerful systems, with power densities that can reach terawatts/cm^2^. These laser systems, with better features and lower prices, offer a high-speed/high-quality tool for laser machining, which is of great interest in both basic and applied research [3,4]. Laser ablation depends on laser wavelength, optical features of laser beam, pulsewidth range, machining method and on the optical-thermal-mechanical properties of the substrate to be processed [5]. Some theoretical descriptions have been developed by many authors to generalize the stages of the ablation process: laser radiation absorption, heat transfer to the target, evaporation and gas-dynamic of the vapor [6,7,8,9,10,11,12,13].

Glass and glass-ceramic substrates are commonly used in a variety of applications such as lasing systems, opto-informatic devices, micro-optical components, mirrors and waveguides [14,15,16,17,18,19]. In particular, glass-ceramic substrates present a great interest for industrial and engineering applications due to their advantages with respect to other ceramic and glassy materials because of their good chemical inertness, high temperature stability and glass transition temperature, low coefficient of linear expansion, excellent thermal shock resistance and superior mechanical properties such as abrasion, impact,* etc.* Furthermore, laser machining and surface modification of these materials are very interesting for functional purposes in industrial applications since these modifications may be applied as thermal-barriers, heat-conductor tracks, inclusion of thermal sensors,* etc.* [20,21,22,23].

In previous works, the formation mechanisms for generating porous layers by pulsed laser irradiation on the surface of a glass-ceramic substrate in function of the laser wavelength and substrate temperature in the nanosecond range were studied [22,23]. In this work we present the wear behavior characterization of this porous layer in order to determine the friction coefficient (COF) and the wear resistance. This knowledge will allow us to compare the tribological behavior in future works since next stages will be to improve the wear resistance by reducing the porosity by means of controlling the atmosphere in which the process is carried out, by a laser cladding treatment on the surface or by injecting Al_2_O_3_ or ZrO_2_ powders into the layer during the laser processing.

## 2. Experimental

### 2.1. Laser Processing

A commercial diode-pumped Q-Switch Nd:YVO_4_ laser system (TruMark 6230, Trumpf) has been used to produce the porous layer. This system operates at a wavelength of 532 nm, Gaussian mode TEM_00_ with a beam quality factor *M*^2^ < 1.2 and a mean power of 7.2 watts. The laser system is equipped with a programmable galvanometer at the output of the cavity controlled by CAD software. In this way, the beam can be deflected making a bidirectional movement in such a way that any predefined pattern and processing procedure can be performed. The machining process is controlled by the diodes pump current *I_P_* (in relation to peak power), pulse frequency *f*, scanning speed *V* and distance between adjacent lines *Δ*. The system incorporates a convex lens with focal length *F* of 160 mm. Thus, using the equations [3]:

(1) $$D_{bw}=\frac{4FM^{2}\lambda }{\pi D_{0}}$$

(2) $$R=\left(\frac{\pi {D_{bw}}^{2}}{4\;M^{2}\lambda }\right)$$

where *D*_0_ is the diameter of the laser beam before the optical lens, the diameter at the focal point *D_bw_* and the Rayleigh range *R* for this system are, approximately, 12 μm and 177 μm respectively.

As material, a glass-ceramic substrate, Ceran Suprema^®^, manufactured by Schott was used. Their chemical composition and structure were fully characterized by the authors in a previous paper [23]. Its main properties are shown in Table 1.

**Table 1:** Properties of the glass-ceramic substrate Ceran Suprema^®^.

Property	Value	Unit
Density	2.5 *	g/cm^3^
Bending strength	110 *	MPa
Knoop Hardness	600 *	–
Thermal conductivity	1.7 *	W/mK
Thermal diffusivity	0.85 ^#^	m^2^/s × 10^−6^
Melting temperature	1300 *	K

* Schott Technical Data; ^#^ Measure carried out at the Institute of Ceramics and Glass.

The sample was placed at focal distance and processed at room temperature. The laser processing was performed with laser pulses of 0.27 mJ, an irradiance of 28 GW/cm^2^, using a working frequency of 20 kHz, a scanning speed of 25 mm/s and a distance between adjacent lines of 10 μm.

### 2.2. Characterization Techniques

Morphology and composition have been determined by means of scanning electron microscopy (SEM) using a JEOL JSM6400 with EDX analysis. Photography has been carried out with a stereoscope microscope.

Critical load and hardness were measured by a Vickers Zwick hardness tester using loads between 0.1 and 5 kgf.

Unidirectional sliding wear tests were conducted using a Microtest tribometer with a ball-on-disk configuration. The tribological pair was formed by the porous glass-ceramic sample and stainless steel (AIS316) or corundum balls (Al_2_O_3_) of 6 and 3 mm diameter, respectively. The normal loads applied ranged from 1 N to 3 N, the contact frequency was 8 Hz, the diameter of the wear track 4 mm and the sliding distance varied between 50 m and 500 m depending on the load and counterbody used in each test. Wear tests were performed at room temperature in the atmosphere of the laboratory. Table 2 gathers wear test conditions used.

**Table 2:** Wear tests conditions.

Test Parameters	Tests 1		Tests 2	
Ball material/diameter, mm	AISI 316/6 mm		Al_2_O_3_/3mm	
Wear track diameter, mm	4		4	
Frequency, rpm/Hz	480/8		480/8	
Sliding speed, m/s	0.1		0.1	
Load, N	3	1	2	1
Sliding distance, m	500	200	50	100

Roughness measurements and wear tracks profile were measured by means of Surface Analyzer 178–821 (Surftest 401) from Mitutoyo. From four profiles measured for each wear track, volume loss was calculated multiplying the average profile area by the length of the circular wear track.

## 3. Results and Discussion

### 3.1. Layer Formation Mechanisms

**Figure 1:**
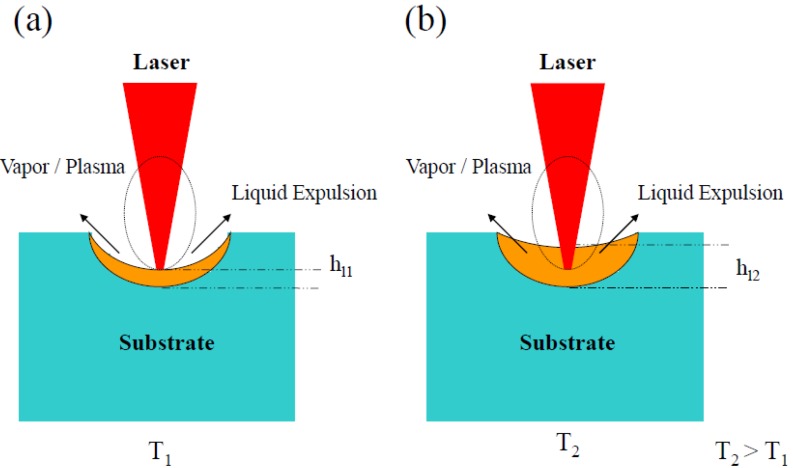
Scheme of ablation process and molten layer of thickness *h_l_* formed in the interaction zone at room temperature *T*_1_ (**a**) and at high temperature *T*_2_ (**b**). The thickness increases when the surface temperature of the glass-ceramic substrate is heated.

The possibility of producing porous layers on the surface of glass-ceramic substrates has been demonstrated when the substrate is machined by means of pulsed lasers in the nanosecond range [22,23]. Laser ablation in the nanosecond range is a photothermal-mechanical process so that the material is removed by the thermal mechanisms activated by the laser beam. They essentially consist of the absorption of the laser energy and subsequent evaporation and ejection of the material. During the ablation process, a thin layer of material in liquid-phase is formed in the interaction zone [2], Figure 1a. The recoil pressure produced in the process squeezes the liquid out from the interaction zone and the material is removed from the surface via evaporation and liquid-phase expulsion. The thickness of liquid phase, *h_l_*, and recoil pressure, *P*_rec_, can be expressed as [2]:

(3) $$h_{l}\propto {\left(D\right)}^{1/2}{\left(\frac{\Delta H_{V}}{I_{a}}\right)}^{1/4}$$

(4) $$P_{\text{rec}}\approx 10^{-5}I_{a}$$

where *D* is the thermal diffusivity, Δ*H_v_* the enthalpy of vaporization, and *I_a_* the absorbed laser intensity.

Since the variations of thermal diffusivity and enthalpy of vaporization with temperature are negligible [24,25], the thickness of the molten layer and the recoil pressure depend mainly on the absorbed irradiance.

**Figure 2:**
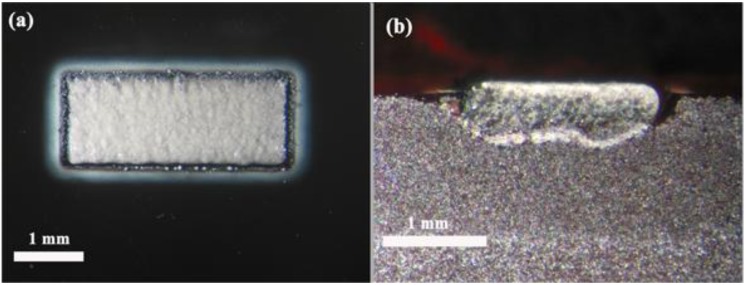
Top view (**a**) and cross-section view (**b**) of the porous layer generated at 600 °C with a laser system emitting at 1064 nm.

The formation mechanisms differ depending on the laser wavelength used. When the laser wavelength is in the near infrared range (NIR) the layer formation depends on the substrate temperature. At room temperature or for temperatures lower than 300 °C the laser processing generates a groove on the surface. Nevertheless, by increasing the substrate temperature, the vibrations of the ions in the lattice also increase. Taking into account that the melting temperature of the glass-ceramic is about 1300 °C, as temperature increases the material acquires a pseudo-plastic character. This behavior together with the fact that the ablated particles ejected from the interaction zone shield the incoming irradiation produce a diminution in the effective irradiance and therefore in the recoil pressure, resulting in an increase of the thickness of the layer in liquid-phase present in the interaction zone, Figure 1b. The pressure exerted by the pulsed laser beam onto the molten interaction zone produces an air bubble generation inside the layer which leads, after cooling, to the generation of the porous layer. Figure 2a,b shows the frontal and cross-section view of the layer obtained by processing at 600 °C. In this case, the porous layer can only be produced at low scanning speeds, 1 mm/s.

The formation of the porous layer is also possible when the laser wavelength is in the green or in the UV range. In this case, the formation mechanisms are in relation with the variation of the absorbance of the glass-ceramic substrate with the wavelength. The absorbance of the glass-ceramics substrate decreases with the wavelength [23]. In particular, the optical absorption coefficients, α, for 355 and 532 nm are 62.13 and 28.37 cm^−1^, 17 and 8 times greater than for 1064 nm, the value of which is 3.52 cm^−1^. As the absorption coefficient increases the absorbed irradiance *I_a_* and the absorbed power per unit of volume (1 − *R*)·α·*I_a_* are higher. Since the values of diffuse reflection *R* for 1064, 532 and 355 nm are 0.94, 0.96 and 0.87 [23], this power density is about 17.52, 31.77 and 68.67 GW/cm^3^ respectively. According to the Equations (3) and (4), when the substrate processing is carried out with a shorter wavelength the pressure increases and a diminution in the thickness of the layer in liquid-phase is produced. Furthermore, the surface temperature can be expressed as [6]:

(5) $${\bar{T}}_{n}≅2T_{m}{\left(\tau_{L}f\right)}^{1/2}$$

where $T_{m}\propto (I_{a}{\left(D\tau_{L}\right)}^{1/2})/k$, $\tau_{L}$ is the pulse width, *f* the working frequency, *D* the thermal diffusivity and *k* the thermal conductivity.

Thus, an increase in the absorbed irradiance produces a higher substrate temperature on the surface, ${\bar{T}}_{n}$. In this way, laser processing at 532 nm or 355 nm produces a local increase in the temperature of the substrate and in the recoil pressure exerted over the substrate, inducing a growth of the crystalline phase of the glass-ceramic substrate and generating the porous layer. Moreover, the increase in the absorption coefficient allows producing the porous layer at higher scanning speeds, 25 and 60 mm/s for 532 and 355 nm respectively. Furthermore, the porous layer can be produced at room temperature, without the drawback of heating the whole sample, and with lower energetic cost, since the energy delivered in the process, calculated by means of the pulse energy, frequency, scanning speed and distance between adjacent lines, are 29.4, 0.86 and 0.42 J/mm^2^ for 1064, 532 and 355 nm respectively. Although laser treatment may cause a build-up of stress in the adjacent regions [26] the features of this laser processing and the porous nature of the layer release the possible stresses produced while the process is carried out, as shown in previous works by means of Raman spectroscopy and mechanical tests [20,27].

**Figure 3:**
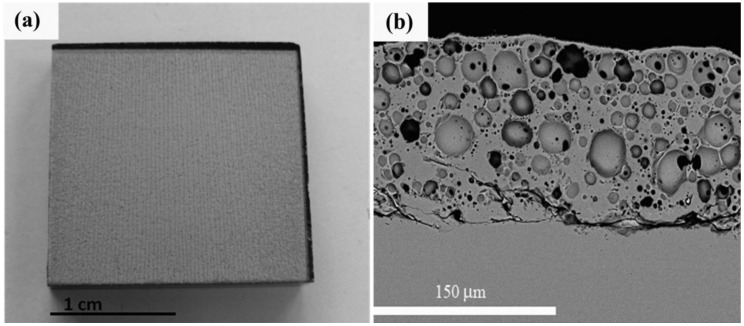
Top view (**a**) and cross-section micrograph (**b**) of the porous coating produced with a laser system emitting at 532 nm.

Figure 3 shows the top view (a) and cross-section micrograph (b) of the porous layer obtained by using a laser system emitting at 532 nm. As seen in Figure 3a the layer presents a texture in the scanning direction produced by the local heating induced by the laser beam. As Figure 3b depicts, the thickness is around 150 μm and presents high porosity.

In this work, the aim is to investigate the wear resistance of the layer. For this purpose, the porous layer was produced by means of a pulsed laser system emitting at 532 nm since the thickness of the layer produced at a wavelength of 355 nm was too thin to assess the wear behavior, and the scanning speed required for producing the coating at a wavelength of 1064 nm was too slow and entails the heating of the whole sample beyond 300 °C.

### 3.2. Compositional and Microstructural Characterization

The chemical composition of the layer was analyzed by semi-quantitative EDX microanalysis, concluding that the composition of the porous layer was the same as the glass-ceramic former and consisted mainly of SiO_2_, Al_2_O_3_, ZnO, MgO and TiO_2_, Table 3, first column. Furthermore a XRD analysis carried out on a laser treated sample and compared to the glass-ceramic substrate showed that the layer was also glass-ceramic. In both cases the crystalline phase was the same, crystals of MgAl_2_Si_4_O_12_, the size of which was around 36 and 42 nm [23].

**Table 3:** EDX Composition of the porous layer before and after wearing tests carried out with AISI316 and Al_2_O_3_ balls.

at. %	Porous Layer	Worn Layer against AISI316	Worn Layer against Al_2_O_3_
**O**	72.10	69.16	72.17
**Mg**	0.51	0.50	0.53
**Al**	6.15	5.74	6.96
**Si**	19.63	16.15	16.45
**Zn**	1.33	0.20	0.48
**Ti**	0.31	0.71	0.41
**Cr**	–	1.15	–
**Fe**	–	6.21	–

### 3.3. Tribological Behaviour

Prior to the wear tests, the hardness of the porous layer was measured to estimate the maximum load that the layer may withstand without cracking during the wear test. Although the nominal hardness of the glass-ceramic Ceran Suprema is 5.56 GPa, due to the porous structure of the layer, loads above 0.4 kgf on the Vickers indenter induce cracking of the layer. Therefore the hardness of the porous layer is remarkably lower than the dense substrate being the hardness measured applying a load of 5 kgf only 1.33 GPa. Therefore, the maximum load that can be applied without severe cracking for wear test is 4 N (0.4 kgf).

**Figure 4:**
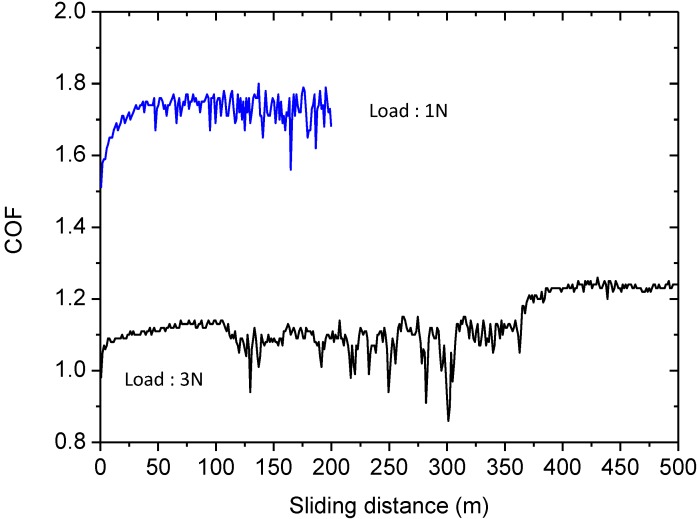
Friction coefficient, COF, recorded during the wear tests against AISI316 ball at 1 and 3 N of normal load with 200 and 500 m of sliding distance, respectively.

Wear behavior was initially tested against AISI316 at two loads, 1 and 3 N. The lower load applies a Hertz initial medium pressure of 0.22 GPa and a maximum pressure of 0.33 GPa, while a load of 3 N applies an hertzian a medium pressure of 0.32 GPa and a maximum pressure of 0.48 GPa. In all cases pressure is below either the measured hardness of the layer or the load limit for cracking. Figure 4 shows the variation of the friction coefficient, COF, with the load. At the lower load, the COF is as high as 1.7 meanwhile at 3 N the COF reduces to 1.1–1.2. In both cases such COF is notably higher than the values reported in the literature for different types of dense glass-ceramic materials, which is around 0.8 for a wide range of loads and counterbodies [28,29,30].

**Figure 5:**
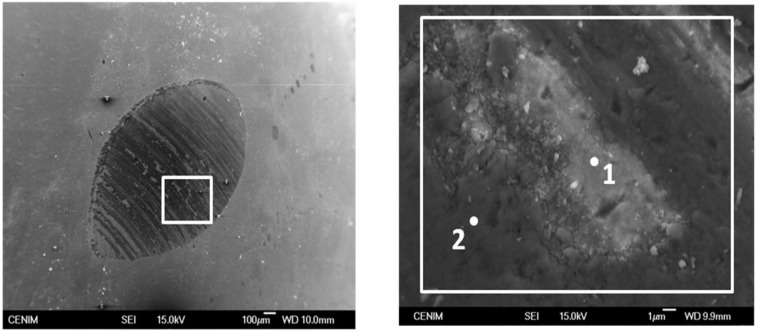
Appearance of the worn surface for the AISI 316 ball showing the areas in which the EDX analyses were carried out (see Table 3).

**Figure 6:**
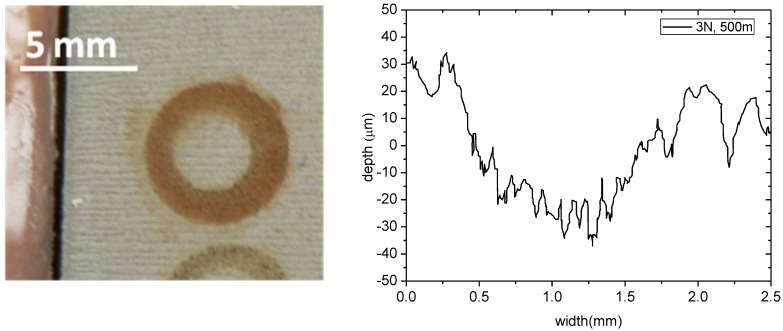
Appearance and profile of the wear track in a glass-ceramic sample after wear test against AISI 316 ball with a normal load of 3 N and sliding distance of 500 m.

The analysis of the worn surfaces revealed that the steel ball appears notably eroded, Figure 5, meanwhile the wear tracks on the glass-ceramic acquire an orange-like color, Figure 6, indicating that oxide transference from the ball to the porous layer has been produced. The analysis of the wear track performed by EDX, confirmed that such material transference occurred between the glass-ceramic layer and the AISI316 ball. Table 3 compares the chemical composition of the porous glass-ceramic layer before and after the wear tests. Similarly Table 4 compares the chemical composition of the ball before and after the same wear test at the different locations identified in Figure 5. While the wear track shows the presence of Cr and Fe, coming from the steel ball, the analysis of the flattened area of the ball shows the presence of Al, Mg, and Si oxides coming from the glass-ceramic material.

The ball exhibits a severe abrasion by the hard ceramic oxide particles and consequently the contact area has increased remarkably during the test. Although the wear track width is very large (around 1.5 mm) the average depth is only 50 μm, Figure 6. This fact indicates that most of the worn volume is concentrated on the AISI316 ball and the apparent width of the wear track is mainly due to the widening of the ball contact area more than a real severe wear of the porous ceramic layer.

In order to reduce the severity of the tests for the steel used as a counterbody, further tests were performed at a lower load and shorter sliding distance, 1 N and 100 m respectively. In these new conditions the glass-ceramic material did not show any measurable volume loss since the wear track is hardly distinguished conversely to what occurred in the steel ball where severe material loss and a wide flattened area is still observed.

**Table 4:** Composition of the AISI316 ball flattened surface in contact with the coating after the wearing test. The analyses were performed in the areas pointed out in the inset of Figure 5b.

at.%	AISI316	Worn AISI316 Ball	
	Ball	1	2
**O**	–	7.85	52.74
**Mg**	–	0.03	0.31
**Al**	–	0.06	2.75
**Si**	–	1.70	7.51
**Ti**	–	–	0.12
**Cr**	14.16	11.41	4.06
**Fe**	85.24	64.97	21.35
**Ni**	0.49	–	–

**Figure 7:**
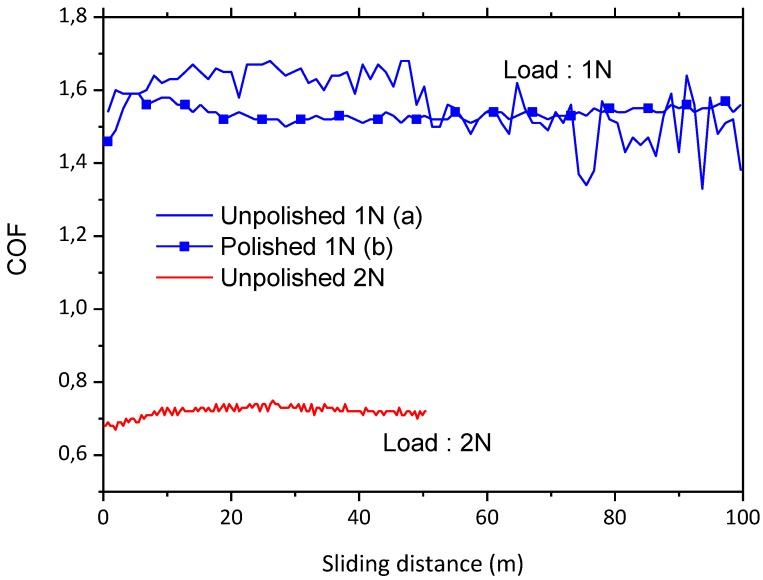
Friction coefficients, COF, for the porous layer against corundum counterbody with the sliding distance for loads of 1 N and sliding distance of 100 m (**a**) unpolished and (**b**) polished samples and for loads of 2 N and sliding distance of 50 m.

In sight of the results obtained from the wear tests carried out using the AISI316 ball, a new batch of tests were performed using a much harder counterbody, namely a corundum ball of 3 mm diameter. Therefore the friction coefficient of the porous layer against corundum was determined for a load of 1 N and sliding distances of 100 m, Figure 7. Although the load employed was the same as in the AISI316 case, the initial Hertz contact average pressure and the maximum pressures were slightly higher, 0.39 and 0.59 GPa, respectively. The friction coefficient recorded is still very high, above 1.4. These values are still significantly higher from those reported in the literature. Cranmer [31] determined the COF values of many commercial glass-ceramic compositions, finding that the average COF values, measured on sample pairs of parallel rings, ranged from 0.49 to 0.7. Similarly, Buchner* et al.* [32] reported values within the same range for various types of ceramics evaluated in a reciprocating sliding configuration against a tungsten carbide ball of 6.3 mm.

In order to discriminate the role played by surface pattern and roughness produced by the laser process the specimens were polished to reduce their roughness. Initially the surface of the porous glass-ceramic layer obtained by laser exhibits a high roughness (average arithmetic roughness Ra) of 6.53 ± 0.67 and 4.90 ± 0.39 μm in the scanning and in the perpendicular direction respectively. The specimens were polished by means of sandpapers and diamond polishing abrasives of up to 1 μm. After such polishing process the laser scans were not visible, and the roughness Ra achieved on the specimens was reduced to 2.65 ± 0.44 μm in any directions. However, wear tests performed on such polished specimens still reveal similar COF values as those found for unpolished samples, Figure 7. Despite the similar COF average value (1.55 approximately) it is clearly observed that the polished sample presents a lower dispersion of data and the COF value is more uniform along the tests. Therefore the high values of the COF recorded in all cases compared to the reported values in the literature, appears to be related to the high porosity of the layer rather than to the roughness.

**Figure 8:**
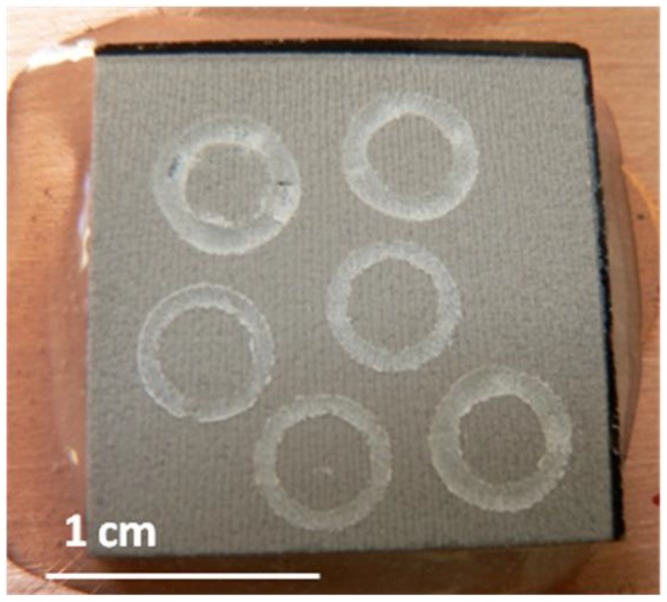
Appearance of the wear track in the unpolished glass-ceramic porous layer performed against corundum balls.

The wear tracks obtained on unpolished samples against corundum balls showed a homogeneous appearance without change of color, Figure 8. The chemical composition of the wear track showed that the composition did not vary from the composition of the porous layer, Table 3, that is to say, no material transference from the ball had been produced. On the other hand, the wear rate calculated is 6.01 × 10^−3^ ± 5.76 × 10^−4^ mm^3^/Nm. These values are higher than those reported by Buchner [32] for common dense glass-ceramic.

The effect of the load in the friction coefficient was assessed by increasing the load to 2 N for a sliding distance of 50 m. In this case the Hertz average and the maximum contact pressure were 0.5 and 0.74 GPa respectively. In these tests a reduction in the COF was observed, as shown in Figure 7, obtaining values around 0.8, in the same order reported in the literature [31,33]. On the other hand, the average wear rate obtained for these tests is 2.85 × 10^−3^ mm^3^/Nm, a value also above typical values in the literature [32].

**Figure 9:**
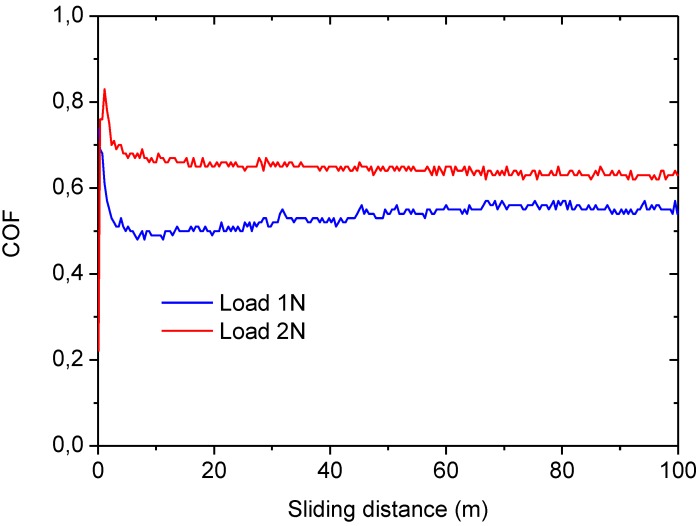
Friction coefficients, COF, for the glass-ceramic substrate against corundum counterbody with the sliding distance for loads of 1 and 2 N and sliding distance of 100 m.

All the results shown above seem to indicate that the porous layer presents a more elevated COF and wear rate than typical dense glass-ceramic materials reported in the literature. This seems not to be a consequence of the high roughness and surface pattern induced by the laser treatment as we have shown this result also in the wear tests on polished samples. In order to discard effects due to the specific glass-ceramic substrate, a wear test on the dense substrate was performed. For that, the porous layer was completely removed by grinding and a wear test with applied loads of 1 and 2 N performed against corundum for a sliding distance of 100 m. In this case, the COF observed, Figure 9, is reduced drastically to values around 0.55–0.65, which is in the range of the reported for other dense glass-ceramic materials. On the other hand, the wear rates measured are in the order of 6.7 × 10^−5^–1.5 × 10^−4^ mm^3^/Nm that are in the same range of the reported for similar glass-ceramic materials. Therefore, the COF and wear rate results found for the porous layer should be attributed to the porous structure itself and not to a compositional or roughness effects.

The results shown from the laser porous layer, polished porous layer and dense glass-ceramic substrate seem to indicate that the generation of the porous structure is the responsible of the increment of COF and wear rate when compared with the values of the dense substrate. It is generally agreed that the presence of porosity is detrimental to the wear resistance of ceramic and composite materials except when the porosity values are low [33]. The pores may act as stress raisers when a load is applied and thus facilitate crack initiation. Such cracks could also easily propagate by connecting pores with high stress concentration, leading to severe surface failure and material removal. Some works on porous titanium dioxide layers [34] and zirconia polycrystalline ceramics [35,36] state that the presence of porosity strongly weakens the resistance of the material during scratching and wears testing. Young’s modulus and hardness of the ceramic structures were substantially reduced with increasing porosity. In general, for a low amount of pores the capability of plastic deformation compared with the dense ceramic is increased. Conversely, greater porosity promoted surface and subsurface cracking. Regarding COF, in crystalline ceramic materials, low to medium porosity did not affect the friction coefficient but at high porosity the friction also increased. High porosity levels lead to the formation and propagation of cracks which favor the occurrence of granular wear particles in the contact area. Cracking is the main factor influencing the wear mechanism of ceramic materials, as a result of its occurrence, friction and wear increased substantially in porous ceramics.

## 4. Conclusions

Porous glass-ceramic layers can be produced on the surface of glass-ceramic substrates when the substrate is machined by means of pulsed lasers in the nanosecond range. Formation mechanisms differ depending on the laser wavelength used. In the NIR range the formation is produced only if the surface temperature is above 300 °C. In this case the formation mechanism is related to the decrease of the recoil pressure and the increase of the thickness of the layer in liquid-phase present in the interaction zone. When the process is carried out at 532 or 355 nm, the formation mechanisms are related to the increase of the absorption coefficient, which leads to a local increase in the recoil pressure and in the temperature of the sample. For these wavelengths the laser processing can be carried out at higher scanning speed and at room temperature, avoiding the heating of the whole sample and with a lower energetic cost. The composition of the porous layer is the same of the former and is made up of the same crystalline phase of the glass-ceramic substrate, MgAl_2_Si_4_O_12_, immersed in amorphous material.

Wear tests carried out against a AISI316 ball counterface gave rise to material transference from the ball to the porous glass-ceramic layer, producing a severe flattening of the ball and only a polishing effect on the porous layer. The friction coefficient observed is substantially higher than for dense glass-ceramic materials.

In contrast, wear tests carried out against a corundum ball showed no material transference between layer and ball and in this case wear volume loss was concentrated in the porous layer. Moreover, wear rate and coefficient of friction also presented higher values than expected for dense glass-ceramic materials.
